# Compliance With Percutaneous Endoscopic Gastrostomy Tube Insertion Guidelines and Associated Complications in a Tertiary Care Setting: A Clinical Audit

**DOI:** 10.7759/cureus.43566

**Published:** 2023-08-16

**Authors:** Muhammad Bilal Ahmad, Farrukh Ansar, Kainaat Shakoor, Muhammad Adnan, Syed Ali Naqi, Zainab Tahir, Mohammad S Rauf, Umair Bin Shafaat Chaudhary, Asad Alamgir, Nabiha Aslam

**Affiliations:** 1 Medicine, Quaid-e-Azam International Hospital, Islamabad, PAK; 2 Surgery, Quaid-e-Azam International Hospital, Islamabad, PAK; 3 Medicine and Surgery, Northwest General Hospital, Peshawar, PAK; 4 Internal Medicine, Fauji Foundation Hospital Rawalpindi, Islamabad, PAK

**Keywords:** patient safety, clinical audit, pegt insertion, complication rates, percutaneous endoscopic gastrostomy tube

## Abstract

Introduction: Percutaneous endoscopic gastrostomy (PEG) tube insertion is a widely utilized enteral access technique offering long-term nutritional support for patients unable to tolerate oral intake. While the PEG tube provides numerous advantages, adherence to evidence-based guidelines is crucial to minimize complications. This study aims to evaluate adherence to PEG tube insertion guidelines and analyze associated complication rates in a tertiary care setting.

Methods: A retrospective clinical audit was conducted at Quaid-e-Azam International Hospital, Islamabad. Data were collected over three years from patients undergoing PEG tube insertion by a single consultant gastrointestinal surgeon. Adherence to guidelines was evaluated using a 10-item checklist developed based on European Society of Gastrointestinal Endoscopy (ESGE) guidelines. Complication rates and patient characteristics were analyzed.

Results: The study included 70 participants (mean age = 72.21 ± 13.17). The PEG tube insertion rate was 100%. The mean checklist score was 8.34 ± 1.2. Laboratory investigations were performed for 98.6% of patients. 91.4% of patients had a life expectancy exceeding 30 days. 60% of patients received an anticoagulation hold. Prophylactic antibiotics were administered to 90% of patients. Psychological counseling and dietician consultation were offered to 38.6% and 64.2% of patients, respectively. Caregivers received specialized training in 98.5% of cases. 12.8% of patients experienced early complications post-procedure, and 14.2% experienced late complications. PEG tube removal occurred in 27% of patients, with only one patient experiencing complications after removal.

Conclusion: Adherence to PEG tube insertion guidelines was observed in various aspects of patient care, resulting in a low incidence of complications. Comprehensive auditing and guideline adherence are essential to ensure optimal patient safety and procedural outcomes.

## Introduction

Percutaneous endoscopic gastrostomy (PEG) tube insertion is a widely employed enteral access technique, offering an effective and safe means of providing long-term nutritional support for patients who are unable to tolerate oral intake [1]. PEG tube insertion represents a prevalent and widely performed medical procedure, with an estimated annual incidence ranging from 160,000 to 200,000 cases in the United States alone [2]. As a minimally invasive procedure, PEG tube insertion has gained prominence due to its potential to improve patient outcomes, enhance quality of life, and reduce healthcare costs compared to surgical alternatives [3]. The growing popularity of PEG tube is attributed to its versatility, as it can benefit a diverse range of patients across various medical specialities. These may include individuals with neurological disorders affecting swallowing function, head and neck cancer patients undergoing radiation or chemotherapy, elderly patients with deglutition difficulties, and those suffering from severe malnutrition or dysphagia-related complications [4]. However, despite its widespread use, PEG tube insertion carries inherent risks, and adherence to evidence-based guidelines is essential to minimize potential complications [5].

To ensure the safe and standardized application of PEG tube insertion, various professional societies have issued guidelines, encompassing patient selection criteria, informed consent processes, pre-procedure preparation, and post-insertion care. The European Society of Gastrointestinal Endoscopy (ESGE) and the American Society for Gastrointestinal Endoscopy (ASGE) are prominent entities that have contributed to formulating such guidelines, focusing on optimizing patient safety and improving procedural outcomes.

Despite its numerous advantages, PEG tube insertion is not without risks, and complications may arise during or after the procedure. Common complications include peristomal wound infection, tube dislodgement, aspiration pneumonia, and gastrostomy site leakage [6]. The complexity of PEG tube insertion lies in the diverse patient population for whom it is indicated. Patients requiring enteral access can present with a wide range of underlying medical conditions, comorbidities, and anatomical variations, necessitating careful consideration of individualized procedural planning and selection of appropriate insertion techniques [7]. As such, the procedure's complexity highlights the significance of adherence to established guidelines to optimize patient safety and outcomes.

Given the ever-increasing demand for PEG tube insertion, adherence to evidence-based guidelines and rigorous auditing of procedural outcomes are paramount to ensure patient safety and optimize the benefits of this procedure. By conducting comprehensive audits and evaluating complication rates, healthcare providers can identify potential areas of improvement, enhance patient care protocols, and develop strategies to minimize adverse events. To address the complexity of PEG tube insertion and the variable rates of complications reported in the literature, this research paper presents the findings of an audit conducted to assess adherence to PEG tube insertion guidelines and to analyze the incidence of associated complications. By evaluating a diverse patient cohort undergoing PEG tube insertion, this study aims to contribute valuable evidence that can inform clinical decision-making, optimize patient care, and improve procedural outcomes.

## Materials and methods

This clinical audit was conducted at Quaid-e-Azam International Hospital, Islamabad, a privately owned tertiary care medical facility. The study design followed a retrospective approach, focusing on all patients who underwent PEG tube insertion by a single consultant gastrointestinal surgeon within the past three years. To ensure a reduction in potential performance bias, the data analysis exclusively involved the cases performed by a single consultant surgeon.

Approval for this research was obtained from the Clinical Audit Department of Quaid e Azam International Hospital, Islamabad. The researchers adhered to the guidelines set forth by the ESGE regarding the endoscopic management of enteral tubes in adult patients [8,9]. To evaluate the adherence to standard practices regarding PEG tube insertion, a comprehensive 10-item checklist was developed after an extensive review of ESGE guidelines. To establish the checklist's authenticity and credibility, three independent consultants critically reviewed and approved it. The pilot study was conducted on 10 record files to further enhance the credibility of the main audit. Subsequently, the approved checklist was implemented to collect data from patient records. The 10-item checklist included the following variables: informed consent, a life expectancy of more than 30 days, psychological preparation, dietician consultation, pre-procedure laboratory investigations, anticoagulation hold, prophylactic antibiotics, pre-op surgical checklist, exclusion of contraindications, and training of carers regarding PEG tube use.

All patients who had undergone PEG tube insertion by the specified consultant surgeon during the previous three years were included in the study. The inclusion criteria for the study required that patients be regularly followed up for a minimum of two years. Any incomplete record files or cases where patients were lost to follow-up were excluded from the analysis.

Data extraction was performed by reviewing the patients' record files, and the information was recorded in a printed questionnaire form. To ensure data reliability and minimize potential errors during extraction and entry, the questionnaire forms were scrutinized by a separate group of evaluators. The confidentiality and anonymity of patients' records were meticulously safeguarded through the utilization of virtual serial numbers during the process of data collection and analysis. It is important to emphasize that no private information was documented in any manner. Upon completing the data collection phase, the responses were imported into IBM SPSS Statistics version 23.0, developed by IBM Inc., Chicago, USA. The variables were adjusted according to their numeric and string properties to facilitate appropriate statistical analysis. Descriptive statistics, including frequencies, means, and standard deviations, were calculated to summarize the collected data.

## Results

The current investigation involved a total of 70 participants, whose demographic characteristics were meticulously examined. The mean age of the cohort was calculated to be 72.21 ± 13.17. The age distribution spanned from 34 to 92 years, with the median age established at 74 years. Within this sample, gender-wise representation was noted, comprising 31 male patients (44.3% of the total) and 39 female patients (55.7% of the overall sample). Furthermore, the 70 presented patients were diagnosed across a diverse spectrum of 11 distinctive medical conditions, as succinctly illustrated in Table 1.

**Table 1:** Baseline Characteristics of Study Participants PEG: Percutaneous endoscopic gastrostomy

Variable	Frequency	Percentage
Gender		
Male	31	44.3%
Female	39	55.7%
Diagnosis		
Cerebrovascular Accident	49	70%
Aspiration Pneumonia	2	2.9%
Parkinson’s Disease	3	4.3%
Epilepsy	2	2.9%
Malignancy with Brain Metastases	4	5.7%
Hypoxic Brain Injury	2	2.9%
Corticobasal Degeneration	1	1.4%
Mucormycosis	2	2.9%
Septic Encephalopathy	2	2.9%
Esophageal Stricture	1	1.4%
Binswanger’s Disease	2	2.9%
Indication for PEG Tube Insertion		
Impaired Swallowing	52	74.2%
Vegetative State	15	21.4%
Nutritional Support due to Anorexia	3	4.3%

In this study, all PEG tube insertions were done using the pull technique. In conformity with the guidelines prescribed by the ESGE, all patients were subjected to a comprehensive process of obtaining written informed consent, which duly considered and excluded any contraindications. Notably, the analysis of the data subsequently revealed that 91.4% (N = 64) of the patients exhibited a life expectancy surpassing 30 days. Prior to the surgical intervention, a recent and mandatory series of laboratory investigations, including complete blood count, liver function tests, international normalized ratio, and activated partial thromboplastin time, were performed for 98.6% (N = 69) of the patients, ensuring a thorough assessment of their health status. Moreover, in accordance with the established ESGE guidelines, 60% (N = 42) of the patients received an anticoagulation hold.

As part of the preparatory measures, prophylactic antibiotics were administered to 90% (N = 63) of the patients, aiming to mitigate potential post-operative infections. Additionally, prior to the procedure, psychological counseling sessions were offered to patients or their attendants in 38.6% (N = 217) of the cases, whereas 64.2% (N = 45) of the patients availed consultation with a dietician, seeking to optimize their pre-operative care. Furthermore, recognizing the crucial role of caregivers in postoperative care, 98.5% (N = 69) of them received specialized training on the proper utilization of the PEG tube, thereby fostering optimal patient care and outcomes. During the post-procedure phase, 12.8% (N = 9) of the patients encountered early complications, whereas 14.2% (N = 10) experienced late complications, as delineated in comprehensive detail in Table 2. These findings provide important insights into the potential challenges and outcomes associated with the intervention under investigation.

**Table 2:** Incidence of Early and Late Complications in the Patients' Cohort PEG: Percutaneous endoscopic gastrostomy

Complications	Frequency	Percentage
Early Complications		
Hemorrhage	3	4.3%
Blockage of PEG Tube	1	1.4%
Persistent Pain at PEG Tube Site	1	1.4%
Purulent Discharge	2	2.9%
Infection at PEG Tube Site	2	2.8%
Late Complications		
Recurrent Infection at PEG Tube Site	3	4.3%
Blockage of PEG Tube	2	2.9%
Buried Bumper Syndrome	2	2.9%
Ulcers at PEG Tube	2	2.9%
Colocutaneous Fistula	1	1.4%

In our study population, 42.9% (N = 30) of the patients adhered to a schedule of regular follow-up, whereas the remaining individuals were subjected to irregular follow-up for a duration of up to 2 years. Subsequently, in 27% (N = 19) of the patients, PEG tube removal occurred at a later stage, and only one patient experienced complications following PEG tube removal. The average score obtained from the checklist assessment was determined to be 8.34 ± 1.2 (Range = 5 - 10).

**Figure 1:**
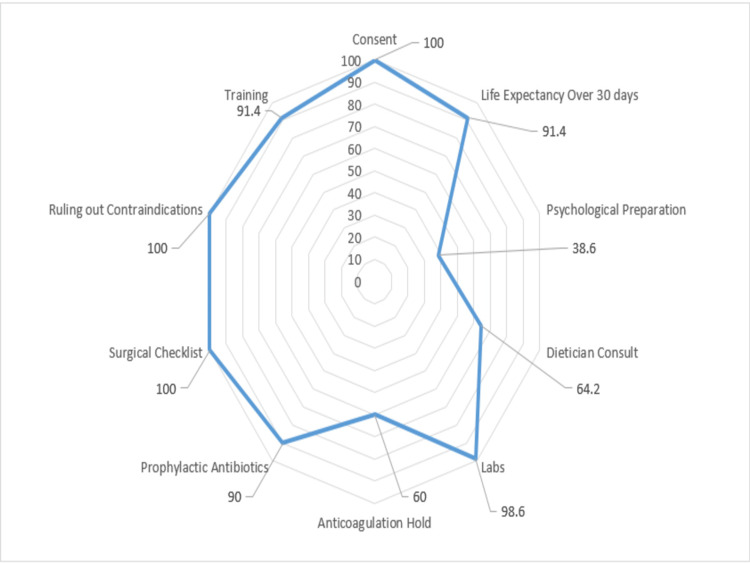
Performance Scores for Checklist Variables

As shown in Figure 1, the strongest adherence was found to be with consent, life expectancy over 30 days, training of PEG tube use, ruling out of contraindications, pre-op surgical checklist, laboratory investigations and administration of prophylactic antibiotics. Identified weak areas were the anticoagulation hold, dietician consult and the psychological preparation of the patients.

## Discussion

Adherence to evidence-based guidelines is crucial to ensure the safety and efficacy of PEG tube insertion procedures. The present clinical audit was aimed to evaluate the adherence to evidence-based guidelines for PEG tube insertion and analyze the incidence of associated complications at a tertiary care hospital in Islamabad. The findings shed light on the practices and outcomes of PEG tube insertion at the hospital and provide valuable insights for improving patient care and procedural outcomes.

The current retrospective study provides valuable insights into the success rate and complications associated with PEG tube insertion. In this study, all PEG tube insertions were done using the pull technique. This technique is widely recommended in clinical practice due to its distinct advantages [9]. The pull technique involves the gradual insertion of the PEG tube from the external abdominal wall into the stomach under endoscopic guidance, facilitating real-time visualization and control throughout the procedure. This method offers superior patient safety by minimizing the risk of inadvertent organ injury, as the endoscopist can meticulously navigate the tube's path, avoiding vital structures [4]. Our findings indicate a 100% insertion rate, which is consistent with previous research conducted by Laasch et al. in the UK [10]. Furthermore, our study demonstrates a superior insertion rate compared to Vanis et al., who reported a technical success rate of 98%, and a study conducted at Karatay University and Medipol University, Turkey, which reported a PEG tube insertion success rate of 97.7% [11,12].

Controlling PEG tube complications is of paramount importance due to its direct impact on patient safety, clinical outcomes, and healthcare costs. Numerous studies have demonstrated that PEG tube insertion is associated with a range of potential complications, including early adverse events such as bleeding, infection, and dislodgement, as well as late complications like tube blockage, gastroesophageal reflux, and stoma site infections. These complications can lead to prolonged hospital stays, increased healthcare expenditures, and, in severe cases, life-threatening conditions [4]. Early complications, occurring during the insertion procedure or within 30 days after, were observed in 12.8% of cases in our analysis. This rate is notably lower than what has been reported in other studies. Boylan et al. reported an early complication rate of 16.7%, while Pih et al. reported a rate of 23.9% [13,14]. Notably, a multi-centre study by Sidorkiewicz et al. revealed an even lower early complication rate of 5.14%, further corroborating the favourable safety profile of PEG tube insertion [15]. Late complications in our study were found to occur in 14.2% of cases, which is considerably lower than the rates reported by Boylan et al. (35.6%) and Pih et al. (26.2%) [13,14]. The most common indication for PEG tube insertion in our patient cohort was central nervous system diseases, accounting for 92.8% of cases. Among these, cerebrovascular accidents represented the predominant diagnosis, constituting 70% of the neurologic causes. Other neurologic indications included Parkinson’s disease (4.3%), epilepsy, hypoxic brain injury, septic encephalopathy, and Binswanger’s disease, each accounting for 2.9% of cases. Malignancy with brain metastases was another neurologic indication, comprising 5.7% of cases (N = 4), and one patient had corticobasal degeneration (1.4%) as the indication for PEG insertion. These findings align with a study conducted at Korea University Ansan Hospital, which reported neurologic causes as the primary indication for PEG insertion in 71.6% of cases [16]. However, in the study mentioned earlier by Pih et al., neurologic indications constituted a lower proportion at 59.9% [14].

Efforts to mitigate complications associated with PEG tube placement are of paramount importance to enhance patient safety and optimize treatment outcomes. Effective strategies encompass an emphasis on procedural expertise and training, ensuring the involvement of skilled and experienced medical professionals proficient in the intricacies of PEG tube insertion. Thoughtful patient selection based on rigorous pre-procedure evaluations, encompassing medical history, nutritional status, and overall health, aids in identifying appropriate candidates, while optimization of patients' health status pre-PEG minimizes risk factors for complications [17]. Strict adherence to infection control protocols during the procedure, coupled with real-time endoscopic guidance to facilitate accurate tube placement and timely identification of potential complications, is crucial for patient safety. Post-procedure care and comprehensive patient education on PEG tube management and recognition of warning signs are integral components in the prevention and timely intervention of complications. The multidisciplinary collaboration among healthcare providers fosters a cohesive approach, where coordinated expertise converges to diminish complications [18]. Ongoing research, refinement of procedural techniques, and adherence to evidence-based guidelines are imperative to further enhance patient safety and minimize PEG-related complications within clinical practice.

Despite providing valuable insights, it is essential to acknowledge certain limitations in our clinical audit. Primarily, the study's retrospective design introduces inherent limitations in establishing causal relationships. Moreover, the focus on a single consultant surgeon's practice at a specific medical facility might restrict the generalizability of our findings to other healthcare settings with different patient populations, procedural approaches, and clinical practices. Patients included in the analysis may not fully reflect the diversity of patients undergoing PEG tube insertion at the hospital, potentially influencing the generalizability of our conclusions. Furthermore, reliance on patient records for data extraction could result in incomplete or missing data, potentially impacting the accuracy and comprehensiveness of our analysis. Incomplete documentation or inaccuracies in medical records could introduce information bias and compromise the validity of our findings.

To address these limitations and enhance the robustness of future research, prospective studies with larger and more diverse patient populations are warranted. Prospective designs can enable the collection of real-time data, allowing for more accurate and comprehensive information on patient characteristics, procedural details, and outcomes. Randomized controlled trials or multi-centre studies involving different healthcare facilities and practitioners would offer more extensive and generalizable evidence regarding PEG tube insertion practices and associated complications.

## Conclusions

This clinical audit in a tertiary care setting demonstrated a high level of adherence to evidence-based guidelines for PEG tube insertion. The study revealed effective pre-operative patient preparation, including thorough laboratory investigations and appropriate anticoagulation holds. Moreover, psychological counseling, dietician consultation, and caregiver training can contribute to optimal post-operative care. The incidence of complications post-PEG tube insertion was relatively low, emphasizing the importance of guideline adherence in minimizing adverse events. The study's findings underscore the significance of conducting regular audits and adhering to established guidelines to ensure standardized, safe, and successful PEG tube insertions. By integrating these insights into clinical practice, healthcare providers can elevate patient care, optimize procedural outcomes, and enhance the overall quality of life for patients requiring long-term enteral access.
